# Defensive and offensive behaviours in a Kleefstra syndrome mouse model

**DOI:** 10.1007/s10071-023-01757-2

**Published:** 2023-03-06

**Authors:** Alejandra Alonso, Anumita Samanta, Jacqueline van der Meij, Liz van den Brand, Moritz Negwer, Irene Navarro Lobato, Lisa Genzel

**Affiliations:** 1 Department of Neuroinformatics, Faculty of Science, Donders Institute for Brain, Cognition and Behaviour, Radboud University, P.O. Box 9010, 6500 GL Nijmegen, The Netherlands; 2 Donders Institute for Brain, Cognition and Behaviour, RadboudUMC, Nijmegen, The Netherlands

**Keywords:** Kleefstra, EHMT1, Autism Spectrum Disorder, Social behavior, Defense, Offense, Aggression

## Abstract

Kleefstra syndrome in humans is characterized by a general delay in development, intellectual disability and autistic features. The mouse model of this disease (*Ehmt1*^±^) expresses anxiety, autistic-like traits, and aberrant social interactions with non-cagemates. To investigate how *Ehmt1*^±^ mice behave with unfamiliar conspecifics, we allowed adult, male animals to freely interact for 10 min in a neutral, novel environment within a host-visitor setting. In trials where the *Ehmt1*^±^ mice were hosts, there were defensive and offensive behaviors. Our key finding was that *Ehmt1*^±^ mice displayed defensive postures, attacking and biting; in contrast, wild-type (WT) interacting with other WT did not enact such behaviors. Further, if there was a fight between an *Ehmt1*^±^ and a WT mouse, the *Ehmt1*^±^ animal was the most aggressive and always initiated these behaviors.

**Supplementary Information:**

The online version contains supplementary material available at 10.1007/s10071-023-01757-2.

## Introduction

Kleefstra syndrome is a neurodevelopmental disorder, which is caused by the haploinsufficiency of the euchromatic histone methyltransferase 1 (*EHMT1*) gene (Kleefstra et al. 2006). This gene is expressed highly in the developing neural system, acting as an epigenetic factor (Shinkai and Tachibana 2011). In humans, this syndrome is characterized by developmental delays, distinct facial features, intellectual disability, and autistic traits (Kleefstra et al. 2006; Vermeulen de Boer et al. 2017). Around 95.7% of Kleefstra patients are diagnosed with autism spectrum disorder (Vermeulen de Boer et al. 2017), characterized by repetitive behaviors, poor verbal social communication, and deficits in social skills. Antisocial behaviors such as anger and emotional outbursts have also been reported in these patients (Kleefstra et al. 2005, 2006; Stewart and Kleefstra 2007; Kleefstra van Zelst-Stams et al. 2009), but these behaviors have not been reported in the mouse model.

The Kleefstra mouse model, *Ehmt1*^±^ mice, reproduces most of the syndrome’s core symptoms (Balemans et al. 2010a). *Ehmt1*^±^ mice have a shorter skull and nose, with their eyes positioned wide apart (Balemans et al. 2014). They have been described as displaying increased anxiety and reduced exploratory drive (Balemans et al. 2010a). Deficits in fear extinction learning, novel object recognition and spatial object recognition have been reported in single-trial experiments (Balemans et al. 2010a). However, in spatial learning studies involving multi-trial experiences and in pattern separation assays, *Ehmt1*^±^ mice can outperform WTs (Benevento et al. 2017; Schut et al. 2020). At the neuronal network level, these mice suffer from hippocampal synaptic dysfunctions, reduced dendritic branching and spine density (Balemans et al. 2010a; Balemans et al. 2014), delay in maturation of parvalbumin GABAergic interneurons in sensory cortical areas (Negwer et al. 2020), and irregular cortical network bursting patterns (Martens Frega et al. 2016), all of which could lead to disabilities in learning and in executive functions such as short term memory and social behaviour.

In human Kleefstra Syndrome patients, caretakers report occasional emotional outbursts in 46.7% of the patients (Haseley Wallis et al. 2021), yet this feature has not been studied in mice. In social settings, *Ehmt1*^±^ mice have been documented to respond in aberrant ways, from an absence of a response to inappropriate or indiscriminate approaches when confronted with unfamiliar mice (Balemans et al. 2010a). In a ten-minute social play session, the time spent socially engaging by two non-cagemates *Ehmt1*^±^ males was on average briefer compared to WT males (Balemans et al. 2010a). To measure sociability, the three-chamber task can be used, where normally WT mice first prefer but then habituate to an unfamiliar mouse placed in one of the chambers. The time spent by WT animals in the chamber with the unfamiliar mouse declines during the latter half of the session. Interestingly in *Ehmt1*^±^ mice this decline is not seen, which was described as persistent behavior by the authors (Balemans et al. 2010a).

**Fig. 1:**
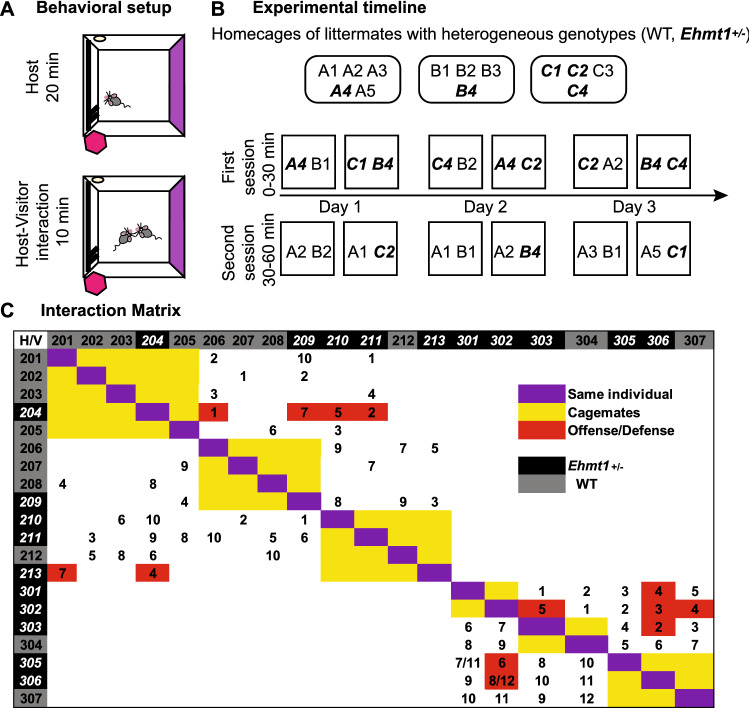
Experimental setup **A**. Task The setup consisted of a 75 × 75 cm × 40 cm square box equipped daily with new intra- and extra-maze cues, as well as a particular smell. The host mouse is placed in a novel environment and is allowed to roam freely for 20 min. After, an unfamiliar, non-cagemate visitor, is introduced to the same environment and left for ten minutes. Colors and stripes inside the maze represent the intra-maze cues, and the pink hexagon represents a 3D extra-maze cue. The yellow circle within the maze represents a scented cotton pad. **B**. Timeline. Example of pairings. On top are depicted three homecages, each consisting of animals WT and *Ehmt1*^±^ (marked in bold italics). In the experimental room, two boxes were available (depicted by squares), and a maximum of 4 trials were performed per day. Counterbalancing was done in a manner that each animal experienced the role of host and of visitor and that they interacted with the same and different genotypes. Some animals had to repeat their roles on different days due to the availability of animals present. The effect of being repeatedly performing in the same role is analysed in Fig. 2E. **C**. Interaction matrix Detail of all interactions. Rows correspond to host (H) and columns to visitor (V), three-digit number corresponds to animal identification. Numbers within the matrix correspond to the day on which the trial took place for that particular cohort. In red are the trials that had defensive or offensive behaviors (color figure online)

In this study, we aimed to further describe the social behavior of the *Ehmt1*^±^ mouse model by using an adaptation of an existing host-visitor task (Granon et al. 2003; Avale et al. 2011; de Chaumont et al. 2012). In Granon’s task, animals were socially isolated for four weeks prior to the task and were allowed to explore a novel box for 30 min. These settings were selected to promote social interaction with a sex- and age-matched visitor which was introduced to the same box for four minutes. No antisocial behaviors among C57BL6/j mice were reported in Granon’s task. In our adapted version of the task, mice were not socially isolated prior to the task and were given 20 min of initial solitary exploration of a novel box (Fig. 1A, top), followed by ten minutes of shared occupancy of the box with a visitor (Fig. 1A, bottom). We performed 74 trials of social interaction among *Ehmt1*^±^ and WT mice, allowing each animal to perform the role of a host and a visitor, and to interact with an animal of the same and different genotype. This study was merely observational and aimed to further describe the model’s phenotype.

## Methods

### Subjects

Ten male *Ehmt1*^+*/*+^ [Wild-Type (WT) c57bl/6j background] and ten male *Ehmt1*^±^ mice were part of this experiment. A power calculation to estimate sample size was not done a priori, since this was a novel experiment and the outcome was not predicted. Animals were bred in-house, 16–20 weeks of age at the start of behavioral training, group housed in heterogenous genotype groups (except one cage, see below) and had ad libitum access to food and water. Mice were maintained on a 12 h/12 h light/dark cycle and tested during the light period. In compliance with Dutch law and Institutional regulations, all animal procedures were approved by the Centrale Commissie Dierproeven (CCD) and conducted in accordance with the Experiments on Animal Act. Project number 2016–014 and protocol number 2016–014-030, approved by the national CCD and local animal welfare body at Radboud University, The Netherlands.

The animals were divided into two cohorts of animals, one composed of three cages with 13 animals (8 WT, 5 *Ehmt1*^±^), and the second cohort of three cages with seven animals (2 WT, 5 *Ehmt1*^±^) (Fig. 1C). All but one cage was composed of mice of both genotypes, creating heterogeneous groups from two to five animals. The difference in a number of mice per cage was due to the fact that all cage mates were also littermates, and to avoid stress by remixing or isolating, the original groups from the in-house breeding facility were maintained. No persistent fighting was observed among cagemates.

### Habituation

All animals were extensively habituated to the experimenters by handling for a period of two weeks before habituation to the experimental environment. By the end of the second week, the animals freely climbed upon the experimenters’ hands and showed no sign of distress when picked up by the experimenters. Pick-ups were performed by the tail until the third day of handling, from there on animals were picked up by cupping (see examples at https://www.genzellab.com/#/animal-handling/).

All mice were habituated to the experimental environment, an empty square box (75× 75 × 40 cm) made of lacquered wood, with no deliberate cues in sight. Habituation was composed of two sessions, in the first of which all animals from the same cage were placed together in the box for 30 min. In the following session, each animal was left to freely roam or explore on its own for ten minutes. The next time the animal would enter the box would be during the experimental phase.

### Experimental environment

Two boxes were used simultaneously in the behavioral room, each box had distinctive colored floors, and every day the cues surrounding and inside the box were changed, meaning that there were two new boxes daily. Pairs of mice would be placed randomly into either of the boxes, and no mouse would ever re-enter the same environment. Cues were composed of everyday materials (paper, fabric, tape) and were attached to the walls inside of the maze in a two-dimensional manner. Walls surrounding the box also were equipped with cues, which were bulkier, to provide three-dimensional cues. Additionally, each novel environment was paired with a particular smell, provided by a cotton pad with scented oil glued within the box, out of the animal’s reach. The smells used were: sandalwood, lavender, vanilla, English rose, jasmine, honeydew, strawberry, blueberry, blue water and musk. All pairs interacting on the same experimental day were cued to the same smell.

Video recordings were achieved with a Logitech HD Webcam C270 clamped onto the ceiling of the experimental room. Light level in the room was ~ 35 lx.

### The social interaction task

Before the beginning of each trial, the floor of the box was thoroughly cleaned with 70% ethanol. The trial began by placing a mouse, the host, in the experimental environment and leaving them to roam freely. Each mouse had already been habituated to the experimental room and to the empty experimental box. At the 20-min mark, a non-cagemate, the visitor, was introduced to the environment, and the host-visitor pair were left to interact for ten minutes (Fig. 1A). This specific pair of animals would not be part of another trial within the same day, but could face each other later in opposite roles on other experimental days (all interactions can be seen in the interaction matrix in Fig. 1C). Each of these animals would also act as host or visitor with different animals in successive days until all animals had experienced both roles (Fig. 1B). Experimenters were blind to the genotype of the animal, and only intervened in case that the host-visitor pair engaged in a fight that resulted in a distress vocalization from one of the mice, in which case the trial was terminated and the animals were placed back in their homecages.

The task used in this research is adapted from Avale et al. (Avale et al. 2011). In Avale’s task, animals were socially isolated for four weeks, and on the day of the experiment, allowed to investigate the novel environment for 30 min. After this period, a visitor mouse was introduced into the environment for four minutes. These conditions were set after observing that non-isolated animals, or with no prior exploration time, preferred to explore rather than interact. In our task, animals were not socially isolated prior, as a pilot of this experiment showed extreme aggressiveness from single-housed animals. Novel box investigation was reduced to 20 min, while social interaction was increased to 10 min. The increased window of time for social interaction was given to allow enough time for the animals to interact, as previous studies had reported that these animals display decreased social interaction (Balemans et al. 2010a).

### Video analysis

Videos were manually scored in duplicate by blinded observers, using hand timers, and the results of both observations were averaged. There was an average difference of 12.9 s (2% difference of total time) for all measures between observers. Behaviors were classified visually, and the three types of behaviors were: sitting, wall and cue exploration, spatial exploration and social interaction. Sitting was defined as when the animal sat grooming or sitting still. Cue and wall exploration was defined as smelling, touching or actively looking into the direction of cues in case they were out of reach. Spatial exploration was defined when the animal was moving throughout the arena and not exploring cues nor sitting. Social interaction was defined as when one or both of the animals were touching or smelling each other.

In the case of trials where there were defensive or offensive behaviors, a defensive-offensive scale was used per individual. The scale had 4 levels, with 0 being no defense, 1 if the animal stands on their hind paws and/or rattles its tail, 2 when the animals engage in a fight, and 3 when the fight reaches a point where one mouse emits a distress vocalization. In this case, the offender is scored with 3 while the offended animal depending on its behavior, is scored from 0 to 2 (if the offended mouse fights back the score would be 2, if it just takes defensive postures the score would be 1, and if the mouse does not defend itself, the score would be 0). Once an animal in a trial reached scored 3, the host-visitor pair were separated by the experimenter and the trial was terminated, to avoid stress and harm to the animals.

In case the trials were terminated, the time at which the trial was stopped was noted, and the percentages of behaviors were calculated out of the total time of the trial.

### Statistical analysis

Statistical analysis was run with each trial as the sample. Analysis was repeated for each animal as the sample in supplementary materials. Since there were trials terminated early, the values used for analysis are percentages of the total time spent in the trial.

All behavioral data were analyzed using IBM SPSS. Data concerning exploratory behavior were analyzed using repeated-measures ANOVAs with time spent in different behaviors as the within-subject variable and genotype (*Ehmt1*^±^ vs. WT) and role (host or visitor) as the between-subject variable. Data concerning social interaction over sessions were analyzed using repeated-measures ANOVA with genotype (*Ehmt1*^±^ vs. WT) and session (1, 2, 3) as the between-subject variables. Data concerning social interaction over sessions for a single genotype were analyzed using univariate analysis with a session as the between-subject variable since not all subjects completed all sessions. Data concerning interaction time in trials with defensive and offensive behaviors and those without were analyzed using independent sample t tests. Posterior analysis was performed using independent sample t tests.

## Results

**Fig. 2:**
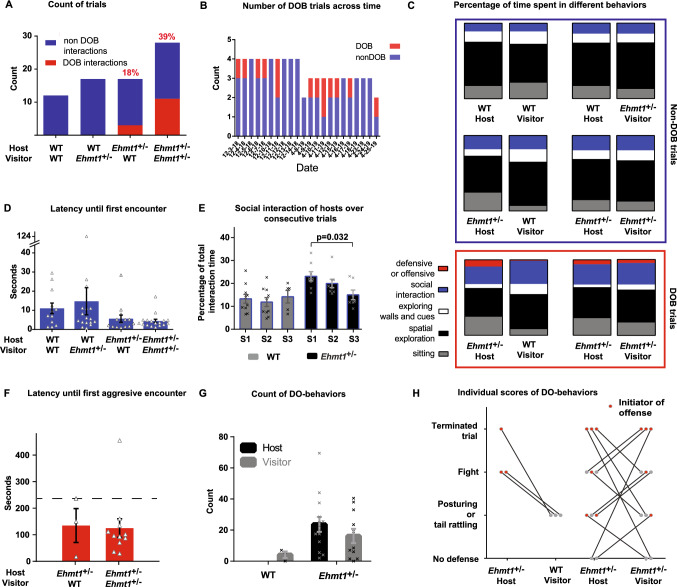
**A** Count of trials with DO-behaviors out of the total number of trials in each type of interaction Red number above indicates the percentage of trials with DO-behaviors within each sample. Legend under each bar represents the genotype of the host-visitor interaction. It is noticeable that defensive and offensive behaviors only occurred if an *Ehmt1*^±^ mouse was present. **B**. Number of DOB interactions in each experimental day. Each day there was a different smell in the behavioral room, and DO-behaviors happened throughout the experimental timeline instead of on a particular day. **C**. Percentage of time spent in different behaviors during the 10 min interaction, organized by host-visitor couples in non-DOB trials (blue square) and in DOB trials (red square). For non-DOB trials visitors explored longer, hosts sat longer and pairings of *Ehmt1*^±^ hosts interacted for longer. In DOB trials, visitors explored longer. There were no differences in interaction time among the pairs, and this is the case for the periods with and without DO-behaviors. When analyzing only the *Ehmt1*^±^ hosts and WT visitors’ pairings, it’s the *Ehmt1*^±^ hosts the ones that spend a longer time in DO-behaviors. For the pairings of *Ehmt1*^±^ hosts and *Ehmt1*^±^ visitors, there is no difference in the amount of time that each spends in DO-behaviors. **D**. Average latency until a first encounter in trials with no DOB interactions Trials with *Ehmt1*^±^ hosts seemed to have a shorter latency to approach the visitor, but this trend was non-significant. **E***.* Average social interaction time of the host during the first 3 sessions (S1, S2 and S3) for trials with no DO-behaviors. Time spent by *Ehmt1*^±^ hosts socially interacting decreased after repeated instances which was not the case for WT hosts. **F**. Average latency until first aggressive encounter Dashed line marks the 4-min mark, which is the length of a trial in the original task. 92% of all trials experienced offense before four minutes. **G**. Average count of DO-behaviors in *Ehmt1*^±^—WT and *Ehmt1*^±^—*Ehmt1*^±^ pairings Results are sorted by the role of each individual. **H**. Individual defensive and offensive scores for host-visitor interactions scale: 0: No defense, 1: Defensive posture (hind paw stand) and/or snake rattle, 2: Fight (biting), 3: Terminated trial due to one of the animals generating a distress sound. Filled circles correspond to values of individual trials, and red filled circles to the animal that initiated the offense. Lines connect each host-visitor pairing. Crosses or triangles correspond to individual trials. Error bar corresponds to SEM (color figure online)

We measured social and explorative behaviors for the duration of the social encounter. *Ehmt1*^±^ and WT mice were combined in all possible configurations, so that each mouse was at least once a host and once a visitor, and interacted at least once with both genotypes (Fig. 1C). Each time an animal entered a trial, it was always in a novel environment. Surprisingly, 14 out of 74 host-visitor interactions showed some level of defensive and offensive behaviors (DO-behaviors) (Fig. 2A), from tail rattling to biting attacks, and 7 out of the 14 trials with such behaviors had to be terminated early due to persistent fighting. 18% of all *Ehmt1*^±^ host – WT visitor trials had DO-behaviors, while in *Ehmt1*^±^ host—*Ehmt1*^±^ visitor trials the incidence went up to 39% (Fig. 2A). Upon analysis it became clear that these behaviors exclusively occurred if there was an *Ehmt1*^±^ mouse in the pair (Fig. 2A) (Chi-Square_3_ = 14.36 *p* = 0.0025), and every *Ehmt1*^±^ animal was part of at least one trial with DO-behaviors. In previous studies using the original version of the task, no antisocial behaviors had been reported among WTs (Avale et al. 2011), and in other studies with *Ehmt1*^±^ mice, no defensive or offensive behaviors have been described (Balemans et al. 2010a).

The different behaviors scored for each animal during the interaction were divided into: time spent sitting, time spent in spatial exploration, time spent exploring the cues and walls, time socially interacting without DO-behaviors and time spent in DO-behaviors (Fig. 2C). To control for the possibility that the smells used daily in the experimental room were not influencing the expression of DO-behaviors, in Fig. 2B we can see a timeline of trials done per day, illustrating how DOB trials happened throughout the experimental timeline, and not on a particular day. The overall incidence of DO behaviors sorted by smell can be found in Supplementary Fig. 1E.

First, we analyze the trials with no DO-behaviors, which were 10 min long, summarized in the blue square in Fig. 2C. The time spent in cue and wall exploration was significantly different for host versus visitors, with visitors exploring longer (F_1,112_ = 4.651, *p* = 0.033) (detailed statistics in Table 1), while hosts sat longer (F_1,112_ = 4.636, *p* = 0.033). The percentage of time spent in social interaction (Fig. 2C, blue blocks) was greater if there was an *Ehmt1*^±^ host (F_1,56_ = 17.159, *p* < 0.001), and there seemed to be an additive effect where there was a linear change from two wild-types to mixed interactions, to finally two *Ehmt1*^±^ spending more time interacting. The latency until the first social encounter showed an opposite trend (Fig. 2D), where the pairings of *Ehmt1*^±^ hosts had a shorter latency to approach an unfamiliar mouse compared to pairings of WT hosts, however, this trend is not significant (F_1,56_ = 3.275, *p* = 0.076).

**Table 1:** Statistical results and key findings Statistical analysis of repeated measures for all tests, except for time spent in DOB for *Ehmt1*^±^*/*WT and *Ehmt1*^±^*/Ehmt1*^±^ pairings, where a *t* test was performed

	Genotype host	Genotype visitor	Role	Gen host*gen visitor	Genotype host*role	Genotype visitor*role	Gen host*gen visitor*role
Non-DOB							
Cue and wall exploration	F_1,112_ = 0.418, *p* = 0.519	Gen_visitor F_1,112_ = 1.521	F_1,112_ = 4.651, *p* = 0.033	F_1,112_ = 1.026, *p* = 0.313	F_1,112_ = 1.764, *p* = 0.187	F_1,112_ = 2.831, *p* = 0.095	F_1,112_ = 0.330, *p* = 0.567
Sitting	F_1,112_ = 1.210, *p* = 0.274	F_1,112_ = 2.186, *p* = 0.142	F_1,112_ = 4.636, *p* = 0.033	F_1,112_ = 0.229, *p* = 0.633	F_1,112_ = 5.121, *p* = 0.026	F_1,112_ = 0.701, *p* = 0.404	F_1,112_ = 7.413, *p* = 0.008
Social interaction	F_1,56_ = 17.159, *p* < 0.001	F_1,56_ = 2.195, *p* = 0.144		F_1,56_ = 0.220, *p* = 0.641			
Latency to approach	F_1,56_ = 3.275, *p* = 0.076	F_1,56_ = 0.084, *p* = 0.773		F_1,56_ = 0.319, *p* = 0.575			

	Genotype host	Session	Gen host*session
Social interaction over sessions	F_1,42_ = 13.194, *p* = 0.001	F_2,42_ = 1.478, *p* = 0.240	F_2,42_ = 2.259, *p* = 0.117
*Ehmt1* ± hosts		F_2,20_ = 4.110; *p* = 0.032	
WT hosts		F2,22 = 0.278, *p* = 0.760	

	Genotype visitor	Role	Genotype visitor*role				
DOB							
Cue and wall exploration	F_1,24_ = 2.802, *p* = 0.107	F_1,24_ = 5.021, *p* = 0.035	F_1,24_ = 0.852, *p* = 0.365				
Social interaction non DOB	F_1,24_ = 0.006, *p* = 0.937	F_1,24_ = 0.252, *p* = 0.620	F_1,24_ = 0.119, *p* = 0.733				
Social interaction with DOB	F_1,24_ = 0.003, *p* = 0.959	F_1,24_ = 3.166, *p* = 0.090	F_1,24_ = 1.473, *p* = 0.237				
	Genotype	Role	Genotype*role				
Count of DOB	F_1,27_ = 6.386, *p* = 0.018	F_1,27_ = 0.54, *p* = 0.818	F_1,27_ = 0.675, *p* = 0.419				

	Key result
Non-DOB	
Cue and wall exploration	Visitors explored longer
Sitting	Hosts sat longer
Social interaction	Social interaction was longer if there was a *Ehmt1* ± host
Latency to approach	Non significative trend. *Ehmt1* ± hosts approach strangers faster
Social interaction over sessions	Social interaction time decreased over sessions
* Ehmt1* ± hosts	Effect observed in *Ehmt1* ± hosts
WT hosts	Effect not observed in WT hosts
DOB	
Cue and wall exploration	Visitors explored longer
Social interaction non DOB	No differences of non-DOB interaction dependent on visitors
Social interaction with DOB	No differences of DOB interaction dependent on visitors
Count of DOB	*Ehmt1* ± perform more DO behaviors

Each animal experienced a maximum of one trial per day, either as a host or a visitor, and on the following days they could perform the task again until every animal experienced the role of host and visitor at least once. To analyze the effect of repeating the task and if their behavior changed over time, we evaluated the time spent by each host engaging in social interaction, split for consecutive trials (Fig. 2E). A trend was noticeable where the amount of time interacting decreased over time in the *Ehmt1*^±^ hosts, and statistical analysis showed an effect of the genotype of the host (F_1,42_ = 13.194, *p* = 0.001). When analyzing the time spent interacting over consecutive trials only for *Ehmt1*^±^ hosts, there was a significant decrease over time (F_2,20_ = 4.110, *p* = 0.032), while when performing the same analysis for WT hosts, no differences could be seen across sessions (F_2,22_ = 0.278, *p* = 0.760).

Overall, in trials with no DO-behaviors, trials with *Ehmt1*^±^ hosts had longer interaction times and shorter latencies until the first social interaction. Over repeated instances of performing the task, the time spent in social interaction decreased for *Ehmt1*^±^ hosts.

Next, we analyze the trials where there were DO-behaviors, which only happened when there was an *Ehmt1*^±^ host (Fig. 2C, red square). The time spent in cue and wall exploration was significantly different for host versus visitors, with visitors exploring longer (F_1,24_ = 5.021, *p* = 0.035). Social interaction in these trials was divided into DO-behaviors and interaction without DO-behaviors. The time spent posturing (standing on hind legs), tail rattling, biting and fighting was scored for each individual of the pairing, as well as every instance of DO-behavior (Fig. 2G). The genotype of the visitor did not influence the time spent socially interacting with or without DO-behaviors. An effect of genotype was found in the counts of all DO-behaviors in both types of pairings (Fig. 2G) (F_1,27_ = 6.386, *p* = 0.018), with *Ehmt1*^±^ animals enacting more DO-behaviors.

Continuing with trials that had DO-behaviors, the latency until the first DO-behavior was not different depending on the genotype of the visitor (Fig. 2F). Increased aggression could have been argued to happen due to the increased interaction time we provided in this adapted version of the task, but in 92% of the time, the first DO-behavior was before four minutes had elapsed, which was the window of time given in the task by Avale et al. (Avale et al. 2011) (dashed line in Fig. 2F), suggesting that the aggressive features observed are not due to an extended period of interaction. Levels of defense and offense were scored using a defensive-offensive scale per individual, as described in the methods (Fig. 2H). Analyzing the defensive-offensive scale values revealed that when there are two *Ehmt1*^±^ animals defensive-offensive levels were similar for host and visitor, but when there is an *Ehmt1*^±^–WT pairing, the *Ehmt1*^±^ was always the biggest offender. The initiator of the offense was also identified in all such interactions (red circles in Fig. 2H), in *Ehmt1*^±^—*Ehmt1*^±^ interactions both the host and visitor were as likely to initiate the first offensive behavior (in 6 instances the initiator was the host, and in 5 instances the initiator was the visitor), while in *Ehmt1*^±^—WT pairings, it was always the *Ehmt1*^±^ animal who initiated the offense.

In sum, DO-behaviors were only observed when *Ehmt1*^±^ animals were hosts. In *Ehmt1*^±^—WT pairings, the *Ehmt1*^±^ mice spent longer in DO-behaviors, had a higher count of DOB and always initiated the offense, while WTs only postured or rattled their tails. In *Ehmt1*^±^—*Ehmt1*^±^ interactions, the visitor spent longer in DOB than the host, but there was no difference in the count of DO behaviors. Both hosts and visitors were likely to initiate an offense, and both roles reached the highest level in our defensive-offensive scale.

## Discussion

The defensive and offensive behaviors observed in our study resemble the emotional outbursts described by caretakers in Kleefstra patients (Haseley Wallis et al. 2021). Previous studies investigating the behavior of *Ehmt1*^±^ mice in social paradigms (Balemans et al. 2010a) have not reported any behavior resembling emotional outbursts. To understand the underlying neurobiological mechanisms affected by Kleefstra syndrome, animal models are crucial. Just as important is the use of adequate behavioral tests that capture key symptoms that may create patient and caregiver suffering. The task used in this study revealed a specific phenotype, which has not been captured in other social tasks: DO-behaviors when confronted with an unfamiliar non-cagemate in a novel environment.

After allowing a host to explore a novel box, an animal from a different cage was introduced for 10 min to interact freely. Since the host had plenty of time to explore the environment, during the interaction period, hosts spent longer sitting while visitors spent longer exploring and no differences were observed due to genotype. These results contradict prior research that attributed decreased walking and increased sitting in *Ehmt1*^±^ animals in an open field setting (Balemans et al. 2010a), however, the differences may be due to the opportunity to explore an environment that had visible cues, and to interact rather than just navigate an empty arena.

The most important findings observed in this study refer to social interaction, both in situations with no defensive or offensive behaviors, and those with. In non-DOB trials, *Ehmt1*^±^ animals interacted for a longer period, which resembles findings where *Ehmt1*^±^ would not decline the amount of time spent next to a novel caged individual but also contradicts results from social play, where *Ehmt1*^±^ spent less time interacting (Balemans et al. 2010a). These differences could be due to differences in the experimental environment, as Avale and colleagues (Avale et al. 2011) have shown that for animals to interact longer they need to explore the environment and be socially isolated for several weeks. In Baleman’s study (Balemans et al. 2010a), animals were isolated for only 30 min prior, and the environment was novel to them, which would promote exploration instead of interaction. The animals in our study had the chance to explore, and once a non-familiar individual was introduced, they quickly approached them and then spent longer than what normally WTs spend with an unfamiliar individual. Social impairments are key symptoms in autism spectrum disorder (Chevallier et al. 2012), and in rodents such impairments can be expressed as a decreased social response or as the opposite, an exaggerated or inadequate response, like the ones we observe here.

In the trials where there were DO-behaviors, these were experienced by every single *Ehmt1*^±^ mouse from our sample, and they only took place when the host was an *Ehmt1*^±^ animal. Several mouse models for autism show social impairments like low social motivation, increased self-grooming, and impaired nest building (Moy et al. 2009) (for a detailed list, see supplementary materials of (Varghese et al. 2017)) while some strains have been reported to show increased aggression or DO-behaviors (Grayton et al. 2013, Burrows et al. 2015). An imbalance in the ratio of excitatory and inhibitory synapses within the cortical, mnemonic and emotional circuits is thought to contribute to the occurrence of diseases with deficits in social behaviors such as autism (Rubenstein and Merzenich 2003; Gogolla et al. 2009; Selten van Bokhoven et al. 2018; Antoine Langberg et al. 2019). DO-behaviors in mice with autistic traces are commonly linked to abnormal response inhibition in neural networks (Grayton Missler et al. 2013; Burrows Laskaris et al. 2015), which have also been reported in the *Ehmt1*^±^ mouse model (Frega Linda et al. 2019; Negwer et al. 2020), and could be underlying the increased DO-behaviors that we report in this current study.

Neural network studies have identified a circuit in which CA2 promotes aggression by activating the lateral septum, which in turn disinhibits the ventromedial hypothalamic subnuclei, directly implicated in aggression (Anderson 2016, Antoine, Langberg et al. 2019). So far, differences in the hippocampus, the dentate gyrus (Benevento et al. 2017), hippocampal regions CA1 and CA3 (Balemans et al. 2014) region, and entorhinal cortex (Gupta-Agarwal et al. 2012) have been reported in WT in *Ehmt1*^±^ mice, but studies focused on CA2, necessary for social recognition, processing the what, when and where of social information (Tzakis and Holahan 2019; Oliva et al. 2020), have not been performed yet.

Different smells could have also influenced DO-behaviors, as the behavioral setup changed daily not only in terms of cues inside and outside the box but also in terms of smells. The sense of smell in rodents influence behavior, with certain smells used as calming agents such as lavender and peppermint (Beakas 2021), and others being aversive, as cat’s smell or spoiled food (Kobayakawa et al. 2007). Trials with DOB happened throughout the experimental timeline, suggesting that there was no particular smell that promoted aggression.

The social interaction task used in our study could be argued to be similar to the resident intruder paradigm, in which aggression has been demonstrated, where an intruder is introduced into the homecage of a sex-matched animal. Previous studies in c57BL/6 animals report different findings, with some not showing aggression in resident intruder tasks (Abramov 2008), while others do (Mertens et al. 2019). This could be a methodology issue, where in some cases the homecage given is clean and the mouse is given 30 min to explore (Abramov et al. 2008) while others don’t change the homecage for several weeks and then introduce the intruder (Mertens et al. 2019). In our case, where animals are introduced to an environment and even though they may have the chance to mark territory, it is also a larger space compared to a homecage, which may influence the WT hosts to not engage in aggressive behavior. WT animals that were part of DOB trials never initiated the offense, and only showed defensive behaviors rather than offensive, while for *Ehmt1*^±^ animals, half of DOB trials had to be terminated early due to persistent fighting. This suggests that the increased aggression observed is not due to the setting of the task, but rather a feature of their phenotype.

Another criticism of our design is the fact that we used the same animals in repeated instances. The main reason for doing this was to reduce the number of animals used in research. To account for repeated instances of experiencing the task over several days, we analyzed the behavior of the hosts over three sessions, which showed us that over time *Ehmt1*^±^ animals tended to interact less with the non-familiar individual. This type of analysis could not be done in trials with DOB due to the small number of instances, but there was no noticeable trend in the frequency and severity of DO-behaviors throughout time. This effect of decreased interaction time over sessions could be due to delayed habituation, which has been shown to be altered in EHMT fly models, as a decreased habituation (Kramer Kochinke et al. 2011).

Using an adapted social interaction task that combines a novel environment with a host-visitor interaction, we were able to show an aberrant social approach and defensive/offensive behaviors in the *Ehmt1*^±^ mouse model for the first time. These findings have two implications. First, they provide a target for trials on potential treatments of emotional outbursts. Second, they show that this task can be used to test for features of defensive and offensive behaviors towards unfamiliar animals in other rodent models of disease.

## Supplementary Information

Below is the link to the electronic supplementary material.

Supplementary file1 (docx 199 KB)

Supplementary file2 (EPS 2253 KB)

## Data Availability

All data analyzed in this study are available from the corresponding author upon request.

## Declarations

### Human and animal participants

This study is reported in accordance with ARRIVE (Animal Research: Reporting of In Vivo Experiments) guidelines.
