# Supplementary material for: Defensive and offensive behaviours in a Kleefstra syndrome mouse model
Source: Anim Cogn. 2023 Mar 6;26(4):1131–40. doi: 10.1007/s10071-023-01757-2 (PMC10345049; doi:10.1007/s10071-023-01757-2)
Supplement: Supplementary file 1 — Supplementary file1 (docx 199 KB) [file 10071_2023_1757_MOESM1_ESM.docx]

*Supplementary materials*


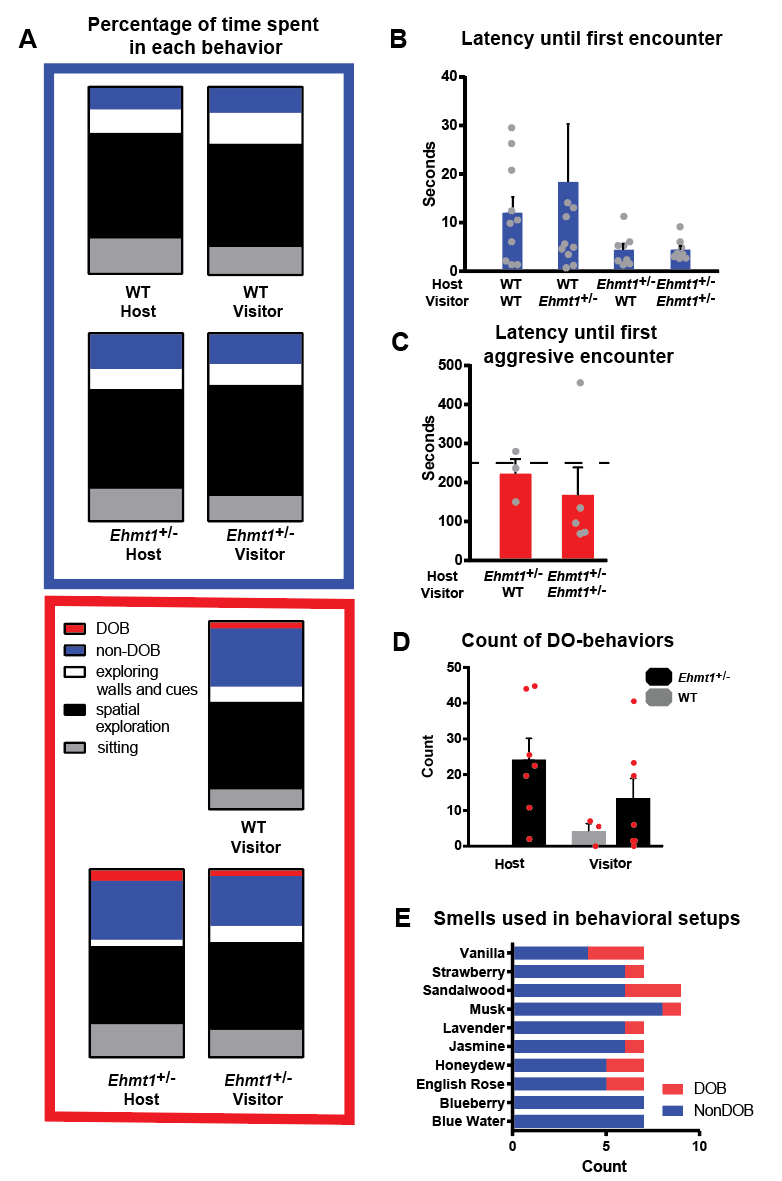


**Supplementary figure 1. Main results shown by animal instead of by trial. A. Percentage of time spent in different behaviors** during the 10-min interaction, divided in non-DOB trials (blue square) and in DOB trials (red square). There were no WT hosts in trials with DO-behaviors, hence there is a column missing. **B. Average latency until first encounter in trials with no DOB interactions** Trials with *Ehmt1^+/-^* hosts seemed to have a shorter latency to approach the visitor, but this trend was non-significant. A value of 124 seconds is out of bounds, for WT- *Ehmt1^+/-^* interactions. **C. Average latency until first aggressive encounter** Dashed line marks the 4-minute mark, which is the length of a trial in the original task. 75% of the animals experienced offense before four minutes. **D. Average count of DO-behaviors in *Ehmt1^+/-^* - WT and *Ehmt1^+/-^* - *Ehmt1^+/-^* pairings. E. Smells used in the behavioral setup.** There was no difference in the incidence of aggression due to a particular smell (Chi-Square_9_=5.054, p=0.7445). Circles correspond to the average of each individual, and error bars correspond to SEM.


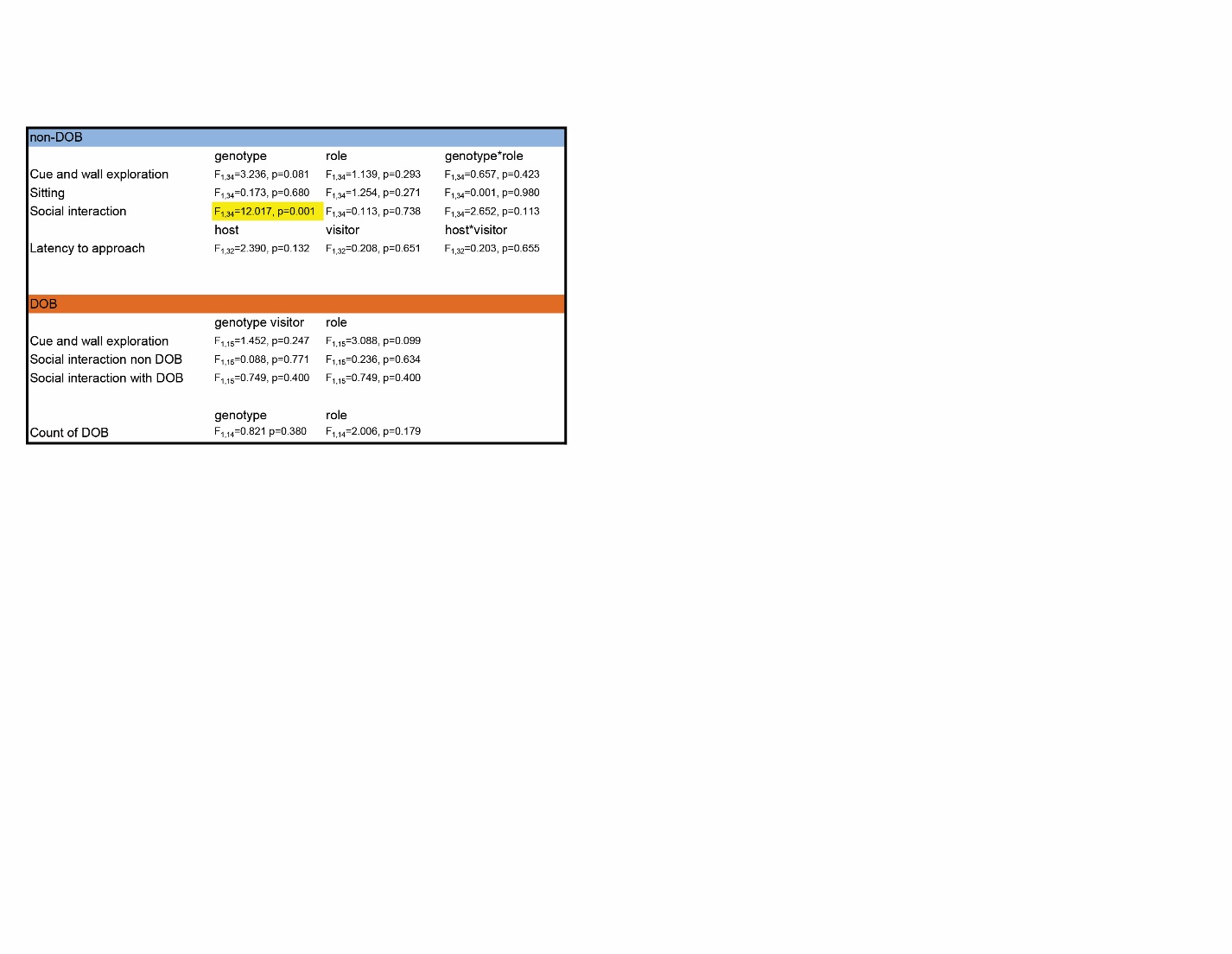


**Supplementary Table 1.** Statistical analysis of repeated measures. In yellow are marked statistically significant results. In this case the key result is that *Ehmt1^+/-^* animals spend longer in social interaction than WT mice.
